# The Effect of Renaltec on Serum Uremic Toxins in Cats with Experimentally Induced Chronic Kidney Disease

**DOI:** 10.3390/vetsci11080379

**Published:** 2024-08-17

**Authors:** Rene E. Paschall, Jessica M. Quimby, Bianca N. Lourenço, Stacie C. Summers, Chad W. Schmiedt

**Affiliations:** 1 Department of Clinical Sciences, College of Veterinary Medicine, The Ohio State University, Columbus, OH 43210, USA; paschall.7@osu.edu; 2 Department of Small Animal Medicine and Surgery, College of Veterinary Medicine, University of Georgia, Athens, GA 30602, USA; lourenco@uga.edu (B.N.L.); cws@uga.edu (C.W.S.); 3 Department of Clinical Sciences, Carlson College of Veterinary Medicine, Oregon State University, Corvallis, OR 97331, USA; stacie.summers@oregonstate.edu

**Keywords:** indoxyl sulfate, p-cresol sulfate, renal disease, Porus One, feline

## Abstract

**Simple Summary:**

In cats with chronic kidney disease (CKD), harmful toxins can build up in their blood, causing health problems. Some of these toxins originate from molecules that are generated in the gut by the action of certain microbes on digested food. Renaltec is a supplement that binds to these molecules in the gut before they enter the bloodstream and are transformed into toxins by the liver. In a study with 13 cats with experimentally induced CKD, researchers tested how well Renaltec worked at reducing two specific toxins, indoxyl sulfate and p-cresol sulfate, in the blood. The cats were given Renaltec either once or twice daily for 56 days, and their toxin levels were measured at different times. The study found that Renaltec significantly lowered the levels of these toxins, with the twice-daily dosing showing better results. This suggests that Renaltec is effective at reducing harmful toxins in cats with CKD.

**Abstract:**

Serum uremic toxins markedly increase in cats with chronic kidney disease (CKD) and have deleterious consequences. Renaltec is an oral adsorbent that binds uremic toxin precursors in the gut. In this prospective cohort study utilizing 13 purpose-bred cats with remnant kidney model-induced CKD (12 IRIS Stage 2, 1 IRIS Stage 3) eating a standardized renal diet, we aimed to assess the effect of Renaltec administration on serum indoxyl sulfate (IDS) and p-cresol sulfate (pCS) concentrations. Cats were sequentially treated with standard of care for 56 days, 500 mg Renaltec orally once daily for 56 days, and then three months later, 500 mg Renaltec orally twice daily for 56 days. Serum IDS and pCS concentrations were measured 28 and 56 days after the administration of Renaltec. Blood pressure and kidney function were measured before and 56 days after the administration of Renaltec. Significant decreases in serum IDS and pCS concentrations were observed for both once- and twice-daily dosing, particularly during the first 28 days of administration. More cats with BID dosing had clinically significant reductions in serum IDS and pCS concentrations than with SID dosing. Renaltec can reduce the serum concentrations of deleterious gut-derived uremic toxins in cats with CKD.

## 1. Introduction

Uremic toxins are deleterious compounds that accumulate with declining kidney function, and thus markedly increase in the systemic circulation of patients with chronic kidney disease (CKD) [1,2,3,4,5]. Currently, there are 146 uremic toxins identified [6]. Amongst the most notable are the gut-derived toxins indoxyl sulfate (IDS) and p-cresol sulfate (pCS). Indoxyl sulfate, in particular, is documented to have deleterious effects on multiple body systems and plays a role in the progression of CKD [7,8,9]. Indoxyl sulfate has been shown to promote renal fibrosis, inflammation, and glomerular sclerosis; induce oxidative stress, and accelerate the senescence of proximal tubular cells; and have adverse effects on erythropoietin production and bone and muscle health, amongst other effects [7,8,9]. Similar to IDS, pCS is documented to have a variety of deleterious consequences, including toxic effects on the kidney and the induction of oxidative stress and inflammation, and has been associated with progression of kidney disease [10,11].

Gut-derived uremic toxins are the metabolites of colonic bacteria which ferment dietary amino acids into uremic toxin precursors [5]. For example, in the case of IDS, dietary tryptophan is converted into indole by gut bacteria in the colon. Indole is then absorbed via the portal system and is further metabolized into IDS by the liver. This waste product is normally excreted into urine; however, with CKD, elimination is compromised [5]. Intestinal dysbiosis and the disruption of the intestinal barrier associated with CKD further exacerbates the production and systemic absorption of gut-derived uremic toxins [2,12,13].

Most uremic toxins cannot be cleared by conventional dialysis due to protein binding. In human medicine, the management of gut-derived uremic toxins consists of a combination of a restricted-protein diet, prebiotics, probiotics, and adsorbents [13,14]. A common strategy is oral adsorbents that bind low-molecular-weight compounds in the intestine. AST-120 is an oral spherical insoluble carbon adsorbent that has been shown to bind indole, the precursor to IDS, and thus reduce serum IDS concentrations in people with CKD, ultimately slowing decline in GFR and prolonging the time until initiation of hemodialysis [1,15,16]. The use of AST-120 at a dosage of 100 mg/kg/day in cats with decreased renal mass demonstrated a dose-dependent reduction in serum IDS concentrations [17].

Renaltec^TM^ (Porus^®^ One, Dechra Veterinary Products, Overland Park, KS, USA) is an oral, 0.1–0.3 mm, smooth, spherical, carbon-based adsorbent that is similar to AST-120 [18]. Renaltec is designed to trap uremic toxin precursors such as indole and p-cresol within pores in the carbon spheres with the aid of opposition electrical charge [18]. Once precursors are trapped by the adsorbent they are excreted with feces, preventing systemic absorption and conversion to IDS and pCS by the liver. Preliminary studies demonstrate that Renaltec decreases serum IDS concentrations in geriatric cats and cats with CKD [19,20].

The aim of this study was to assess the effect of the oral administration of Renaltec on serum IDS and pCS concentrations in cats with experimentally induced CKD fed a standardized diet. The secondary aim was to evaluate for renal biochemical changes after receiving Renaltec. We hypothesized that Renaltec would reduce serum IDS and pCS concentrations in cats with experimentally induced CKD, exceeding the biological variability of these toxins for some cats. Secondarily, we hypothesized that renal clinicopathologic parameters would remain stable.

## 2. Materials and Methods

### 2.1. Study Cats

Thirteen purpose-bred research cats with experimentally induced CKD were enrolled in the study [21]. The cats were obtained originally from a commercial source (Marshall Bioresources, North Rose, NY, USA) and the remnant kidney model was performed for the purposes of a previous study (4 years prior). The current study was approved by the Institutional Animal Care and Use Committee at the University of Georgia (Animal Use Protocol-A2022 10-002-Y2-A13). The cats were housed at the University of Georgia in an indoor vivarium with a controlled environment. The cats were housed and fed individually in USDA-approved cages with food and water bowls, an elevated shelf or hammock, a litter box, and toys for environmental enrichment. Caloric intake for the cats was calculated utilizing https://petnutritionalliance.org/resources/calorie-calculator?type=cats (accessed on 1 May 2023) and was not changed throughout the study.

At enrollment in this study, the cats (8 spayed females and 5 neutered males) were all 5 years old. Twelve cats were staged as International Renal Interest Society (IRIS) Stage 2 CKD and one cat was staged as IRIS Stage 3 CKD according to current guidelines [22]. Six cats received amlodipine ranging from 0.625 mg to 2.5 mg once a day for the treatment of systemic arterial hypertension. When appropriate, amlodipine was administered two hours after the administration of Renaltec. Three cats received 100 mL Lactated Ringer’s subcutaneous fluids 2–3 times per week. One of these three cats also received potassium chloride in their subcutaneous fluids. No new medical interventions were implemented during the study period. All cats were fed a commercial therapeutic diet formulated for cats with kidney disease (Hill’s Prescription Diet k/d dry with chicken during SOC and SID trial, Hill’s Prescription Diet k/d, ActivBiome+ Kidney Defense with oceanfish during BID trial) and their daily food intake was recorded.

### 2.2. Study Design

**Figure 1:**
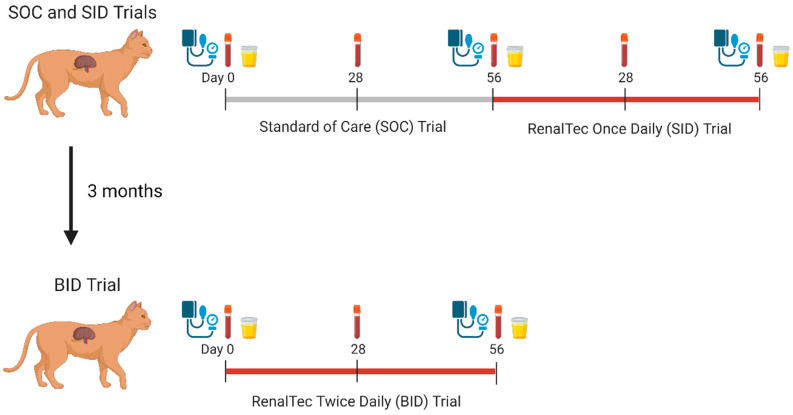
Study timeline and procedures.

The study was conducted as three separate consecutive trial periods. In the first trial, cats were monitored while receiving standard of care (SOC) for 56 days (SOC Trial). In the second trial, which started immediately after the first, Renaltec (500 mg) was administered once a day (SID) in liquid cat snack immediately prior to a meal for 56 days (SID Trial). In the third trial, Renaltec administration was performed twice a day (BID) in liquid cat snack immediately prior to a meal for 56 days (BID Trial) (Figure 1). There were 3 months between trials 2 and 3 during which the cats did not receive Renaltec. During all trial periods, the cats continued to receive SOC.

### 2.3. SOC and SID Trials

All 13 cats participated in the SOC and SID Trials. During these periods, all cats were fed once a day in the morning. The cats were given a liquid cat snack (Churu, Inaba, Torrence, CA, USA) once daily starting one month prior to the initiation of the SOC Trial and continuing throughout the SOC and SID Trial periods. Renaltec was mixed into the liquid cat snack during SID and BID trials. This was given separately from medications. Cats had a full physical exam performed by a veterinarian, including body weight, body condition score (BCS; scale of 1–9), and muscle condition score (MCS; normal = 1, mild loss = 2, moderate loss = 3, and severe loss = 4) once monthly. Blood collection for measurement of serum IDS and pCS concentrations was performed once monthly (SOC Day 0, SOC Day 28, SOC Day 56, SID Day 28, and SID Day 56). Blood pressure (BP) by Doppler sphygmomanometry, complete blood cell count (CBC), serum biochemistry, urinalysis, and urine protein–creatinine ratio (UPC) were measured at SOC Day 0, SOC Day 56, and SID Day 56. As the SID Trial started immediately after the SOC Trial, SOC Day 56 served as the Day 0 time point for the SID Trial (Figure 1).

### 2.4. BID Trial

Only twelve cats participated in the BID Trial as during the three months between the SID and BID Trials, one IRIS Stage 2 female cat was humanely euthanized due to a uremic crisis that failed to respond to supportive care. During the BID Trial period, cats were fed BID by dividing their daily ration into two portions. Cats received 500 mg of Renaltec mixed into their liquid cat snack BID for 56 days. Physical examination and blood collection for the measurement of serum IDS and pCS concentrations were performed on BID Day 0, BID Day 28, and BID Day 56. Blood pressure, CBC, serum biochemistry, urinalysis, and UPC were performed at BID Day 0 and BID Day 56 (Figure 1). Due to a formulary change in the commercial therapeutic diet, cats in the BID Trial phase were inadvertently fed a different formulation of the diet starting one week prior to the start of the trial that included a prebiotic fiber complex (Hill’s Prescription Diet k/d, ActivBiome+ Kidney Defense with oceanfish). The nutritional content of the two commercial cat foods fed during the study is found in Table 1.

### 2.5. Sample Collection and Processing

For logistical ease, the cats were split into two cohorts to allow staggering of days when physical examination and sample collection were performed. The time of day of blood collection was standardized throughout the study. To palliate anxiety, cats were accustomed to venipuncture procedures utilizing positive reinforcement and feline-friendly handling techniques. In the SOC Trial, ancillary sedation (0.2 mg/kg butorphanol IM) was necessary in 3 cats at one study time point (SOC Day 0). In the BID Trial, gabapentin (5–7.5 mg/kg PO) was administered to all cats 2 h prior to venipuncture. Blood pressure was obtained using Doppler sphygmomanometry following a period of acclimation and prior to the administration of any anti-anxiety medication or sedation; 10 measurements were recorded, the first was discarded, and the remaining 9 averaged together. CBC, serum biochemistry, urinalysis, and UPC were performed by the clinical pathology laboratory at the University of Georgia. Blood samples were collected in a glass tube with EDTA (BD Microtainer) for CBC which was performed using Siemens Advia 2120i with manual microscopic differential. The remaining blood was collected in a glass tube (Monoject) with no anticoagulants. Samples were either immediately submitted to the clinical pathology laboratory without centrifugation, or allowed to clot for 30 min and centrifuged (2500 rpm for 15 min) depending on the time of sample collection. For the centrifuged samples, serum was removed, placed in a new glass tube, refrigerated, and submitted the following day. Serum biochemistry was performed using Roche Cobas c501. The remaining serum was aliquoted, frozen, and stored at −80 °C until analysis for IDS and pCS. Batched serum total IDS and pCS concentrations were measured by liquid chromatography–tandem mass spectrometry by the Analytical Resources Core—Bioanalysis and Omics (ARC-BIO) at Colorado State University as previously described [2]. Samples for urinalysis and UPC were collected via cystocentesis using a 22-gauge needle. Urine was submitted to the clinical pathology laboratory in a glass tube. The urinalysis was performed using a combination of the manual inspection of dipsticks (Siemens Multistix) and specific gravity (Reichart TS meter), and a manual microscopic sediment exam. UPC was performed using Roche Cobas c501.

### 2.6. Statistical Analysis

Statistical analysis was performed using Prism software (Prism 9, Prism Graphpad IN., La Jolla, CA, USA). All data were tested for normality with a D’Agostino and Pearson test. As not all data were normally distributed and the sample size was small, nonparametric tests were utilized for analysis. Descriptive statistics were performed for all measurements. For all three trials, serum IDS and pCS concentrations, weight, BCS, MCS, BP, UPC, and select CBC and serum biochemistry values pertinent to CKD were analyzed with a Friedman test. The average pre-Renaltec (SOC Day 0 to SOC Day 56) vs. average post-Renaltec (SID Day 28 and SID Day 56) administrations of IDS and pCS were analyzed with Wilcoxon test. The percent change in serum IDS and pCS concentrations at Days 28 and 56 for both the SID and BID Trials was determined by the formula [final concentration–starting concentration/starting concentration]. The percent changes in serum IDS and pCS concentrations during SID and BID dosing were compared with the Wilcoxon test. The reference change value for IDS and pCS calculated using biological variation estimates has previously been determined in cats using the same methodology for analysis [23]. The number of cats in each trial that achieved a decrease in IDS serum concentration of at least 21.9% and a decrease in pCS serum concentration of at least 28.9% were compared with Fisher’s exact test [23]. Significant weight loss was defined as >5% loss from baseline and determined by the formula [current weight-baseline weight/baseline weight]. A value of *p* <  0.05 was considered significant for all analyses.

## 3. Results

### 3.1. Weight, Body Condition Score, Muscle Condition Score

During the SOC and SID Trials, every cat consumed 100% of its diet every day during the study. The median (range) data for weight, BCS, and MCS are presented in Table 2. When the entire 4 months of the trial was considered, there was a significant decrease in weight between SOC Day 0 and SID Day 28 (*p* = 0.007) and SOC Day 0 to SID Day 56 (*p* = 0.02). There were no significant changes in BCS or MCS during these time periods. When just the 2 months during which cats received Renaltec were considered, no significant changes in body weight, BCS, or MCS were observed. When considered individually, five cats lost significant weight (>5% loss) during the entire 4-month trial. However, only one cat lost significant weight during the Renaltec administration period. During the BID Trial, every cat consumed 100% of its diet at each meal. The median (range) data for weight, BCS, and MCS are presented in Table 3. There were no significant changes in weight, BCS, or MCS throughout the 2-month study.

### 3.2. Clinicopathologic Parameters

Table 2 displays the clinicopathological parameters pertinent to kidney disease obtained during the SOC and SID Trials. When data from the entire 4 months of the SOC and SID Trials were assessed, there was a significant increase in serum creatinine concentrations between SOC Day 0 and SID Day 56 (*p* = 0.0006) and between SOC Day 56 and SID Day 56 (*p* = 0.04). There was a significant increase in BUN concentrations between SOC Day 0 and SID Day 56 (*p* = 0.01) and SOC Day 56 to SID Day 56 (*p* = 0.004). There was an overall significant increase in serum bicarbonate concentrations from SID Day 0 to SID Day 56, but this did not reach significance on post hoc analysis (*p* = 0.06). There was a significant decrease in the anion gap between SOC Day 0 and SID Day 56 (*p* = 0.01) and between SOC Day 56 and SID Day 56 (*p* = 0.007). There was no significant difference in blood pressure. For blood pressure, the results were similar when cats receiving amlodipine were excluded from the analysis.

Table 3 displays the clinicopathological parameters pertinent to kidney disease obtained during the BID Trial. BUN concentrations significantly increased from BID Day 0 to BID Day 56 (*p* = 0.0005), but there was no significant difference in serum creatinine concentrations. Serum phosphorous concentrations also significantly increased from BID Day 0 to BID Day 56 (*p* = 0.001). Serum bicarbonate concentrations significantly increased from BID Day 0 to BID Day 56 (*p* = 0.02). Hematocrit significantly decreased from BID Day 0 to BID Day 56 (*p* = 0.01). There was no significant difference in blood pressure.

### 3.3. Serum IDS and pCS Concentrations

**Figure 2:**
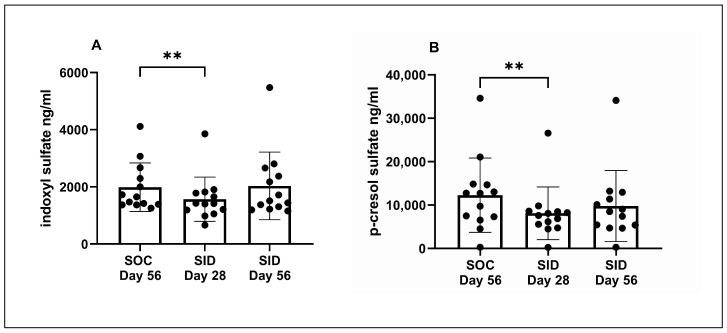
Serum indoxyl sulfate (**A**) and p-cresol sulfate (**B**) concentrations at Day 56 of standard of care (SOC) and after 28 and 56 days of once-daily (SID) Renaltec administration. Each dot represents an individual cat. ** *p* ≤ 0.01.

Table 2 displays the uremic toxin serum concentrations obtained during the SOC and SID Trial periods. When serum IDS and pCS concentrations were compared between SOC Day 0, SOC Day 28, and SOC Day 56, no significant difference in IDS or pCS was observed across the three time points. After daily administration of Renaltec, the serum concentrations of IDS and pCS significantly decreased at SID Day 28 compared to SOC Day 56 (*p* = 0.01 and *p* = 0.003, respectively) (Figure 2A,B). No significant changes in either IDS or pCS concentrations were observed at SID Day 56 relative to SID Day 28 or SOC Day 56.

**Figure 3:**
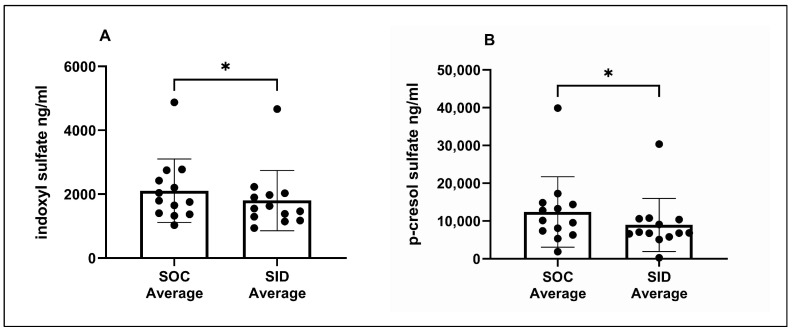
Average serum concentrations of indoxyl sulfate (**A**) and p-cresol sulfate (**B**) during the 56-day standard of care period prior to Renaltec administration versus average serum concentrations over 56 days of once-daily (SID) Renaltec administration. Each dot represents an individual cat. * *p* ≤ 0.05.

When serum concentrations from the SOC period (SOC Days 0, 28, and 56) and post-Renaltec (SID Days 28 and 56) were averaged, the averaged serum IDS (*p* = 0.04) and pCS concentrations (*p* = 0.01) during Renaltec administration were significantly decreased relative to the averaged concentrations during the SOC period (Figure 3A,B).

**Figure 4:**
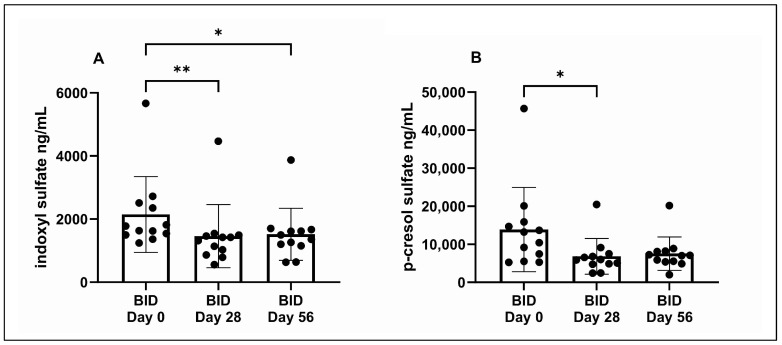
Serum indoxyl sulfate (**A**) and p-cresol sulfate (**B**) concentrations at baseline (day 0) and after 28 and 56 days of twice-daily (BID) Renaltec administration. Each dot represents an individual cat. * *p* ≤ 0.05. ** *p* ≤ 0.01.

Table 3 displays the uremic toxin serum concentrations obtained during the BID Trial. After BID administration of Renaltec, serum IDS concentrations significantly decreased at BID Day 28 (*p* = 0.002) and BID Day 56 (*p* = 0.02) relative to BID Day 0 (Figure 4A). Serum pCS concentrations significantly decreased at BID Day 28 (*p* = 0.02) but not at BID Day 56 (*p* = 0.07) relative to BID Day 0 (Figure 4B).

### 3.4. Percent Change in Uremic Toxins

The median percent change in serum IDS concentrations at Day 28 was −6.4% (range −59–78.6%) during the SOC Trial, −7.1% (range −67.1–39%) during the SID Trial, and −27% (range −77.8–4.3%) during the BID Trial. The median percent change in serum IDS concentrations at Day 56 was −4.2% (range −46.7–169%) during the SOC Trial, −8.5% (range −75–86.3%) during the SID Trial, and −30.5% (range −75–86.3%) during the BID Trial. The median percent change in serum pCS concentrations at Day 28 was 7.7% (range −34.7–129.3%) during the SOC Trial, −29.5% (range −70.6–75.9%) during the SID Trial, and −49.2% (range −87.7–13.2%) during the BID Trial. The median percent change in serum pCS concentrations at Day 56 was 2.1% (range −46.3–148.5%) during the SOC Trial, −16.5% (range −64.9–162.8%) during the SID Trial, and −47% (range −70.8–126.1%) during the BID Trial.

**Figure 5:**
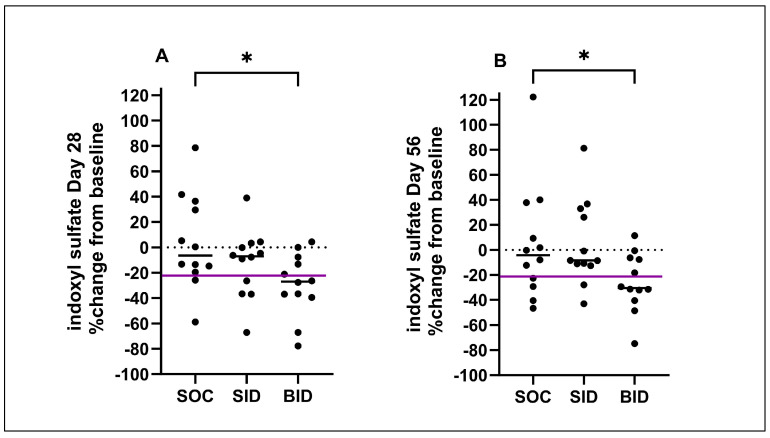
Percent changes in serum indoxyl sulfate concentration (IDS) during the standard of care period (SOC), and during the 28-day (**A**) and 56-day (**B**) administration of Renaltec either once (SID) and/or twice daily (BID). The purple line indicates the reference change value for serum IDS concentrations in cats representing a clinically significant reduction [23]. Each dot represents an individual cat, the solid black line represents median percent change and the dashed line represents zero percent change. * *p* ≤ 0.05.

**Figure 6:**
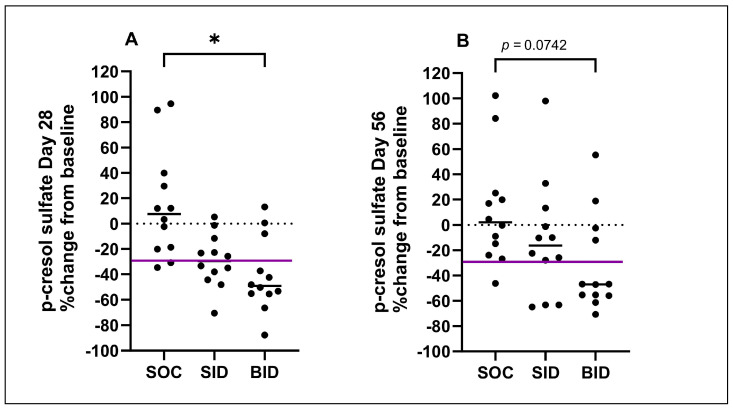
Percent change in serum p-cresol sulfate (pCS) concentration during standard of care period (SOC), and during the 28-day (**A**) and 56-day (**B**) administration of Renaltec either once (SID) and/or twice daily (BID). The purple line indicates the reference change value for serum pCS concentration in cats, representing a clinically significant reduction [23]. Each dot represents an individual cat, the solid black line represents median percent change and the dashed line represents zero percent change. * *p* ≤ 0.05.

When the percent changes in serum IDS and pCS concentrations during the SOC Trial, SID Trial, and BID Trial were compared, there was a significant difference in the percent changes in serum IDS concentrations between the SOC Trial and BID Trial after both 28 and 56 days (Figure 5). There was also a significant difference in the percent changes in serum pCS concentrations between the SOC Trial and BID Trial at 28 days (Figure 6). There was no significant difference in the percent changes in serum IDS or pCS concentrations between the SID Trial and the SOC Trial, or between the SID and BID Trial.

The percent change in serum IDS and pCS concentrations during the 28- and 56-day dosing periods for each individual cat was compared to published reference change values (IDS RCV −21.9%; pCS RCV −28.9%) for these toxins [23]. At Day 28 of BID dosing, 58% of cats had a greater than 21.9% decrease in serum IDS concentrations compared to 33% with SID dosing. At Day 56 of BID dosing, 58% of cats had a greater than 21.9% decrease in IDS compared to 17% with SID dosing. At Day 28 of BID dosing, 75% of cats had a greater than 28.9% decrease in pCS compared to 50% with SID dosing. At Day 56 of BID dosing, 67% of cats had a greater than 28.9% decrease in pCS compared to 25% with SID dosing. When Fisher’s exact test was performed, the number of cats with clinically significant reductions in serum IDS and pCS concentrations between SID dosing or BID dosing was not significant at either time point.

## 4. Discussion

This study aimed to assess the effect of the administration of Renaltec on serum uremic toxin concentrations in cats with experimentally induced CKD eating a standardized diet. This study found significant decreases in serum concentrations of IDS and pCS for both SID and BID dosing after 28 days of administration. Only BID administration resulted in a significant reduction in serum IDS concentrations at day 56. There was no significant difference in IDS and pCS serum concentrations between the data points during the SOC Trial and these data help strengthen the findings that uremic toxins decreased after administration of Renaltec.

When the changes in IDS and pCS serum concentrations for individual cats were compared to published reference change values [23], a majority of cats had a clinically significant reduction in IDS and pCS concentrations after 28 days of receiving Renaltec at BID dosing. While there was no statistically significant difference in the proportion of cats with a clinically significant reduction in serum IDS and pCS concentrations between SID and BID dosing, more cats with BID dosing had clinically significant reductions in serum IDS and pCS concentrations than with SID dosing. While SID dosing may be beneficial in some cats, the success in achieving a clinically significant reduction in serum IDS and pCS concentrations was higher with BID dosing. It is unknown whether the differing feeding habits of cats may affect efficacy as some cats may eat many small meals throughout the day as opposed to being portion-fed two main meals. The significance of the timing of the administration of Renaltec in reference to a meal is also unknown. However, collectively, our data support that the daily administration of Renaltec can reduce serum concentrations of deleterious gut-derived uremic toxins in cats with CKD.

These findings are similar to previous preliminary studies in cats. The effect of the administration of Renaltec on serum IDS concentrations was assessed in 18 apparently healthy geriatric cats (ages 11–18 years) [19]. Cats were divided into two groups: 12 cats received 500 mg Renaltec in a liquid cat snack once a day for 56 days, and 6 cats served as controls. After the administration of Renaltec, the average serum IDS concentration decreased significantly from 1637.6 mg/L at baseline to 650 mg/L at Day 56 (*p* = 0.006). The control group had no significant change in IDS levels over the course of 56 days (average 323.8 mg/L at baseline versus 599.7 mg/L on Day 56) [19]. The administration of Renaltec was also assessed in 19 client-owned cats with stable IRIS Stage 2 and 3 CKD receiving SOC [20]. Cats were randomized to the Renaltec group (n = 10) or SOC (n = 9) and followed for 6 months. The serum concentrations of IDS and clinicopathological parameters were assessed at baseline and 3 and 6 months. At 6 months, cats who received Renaltec had significantly lower serum concentrations of IDS and lower UPC than cats in the control group. One cat in each group progressed from Stage 2 to Stage 3 CKD, and one cat in each group experienced a >25% increase in serum creatinine without progressing in IRIS Stage [20]. Cats in the control group had significantly lower hematocrit and body weight at 6 months compared to baseline. It was concluded that the administration of Renaltec would be of benefit to cats with IRIS Stages 2 and 3 CKD [20]. No previous studies have assessed the effect of Renaltec administration on pCS in cats.

In our study, SID administration of Renaltec appeared to decrease uremic toxins most effectively at 28 days, but not at 56 days. In contrast, the 6-month-long field study demonstrated that cats who received SID Renaltec had significantly lower serum concentrations of IDS than cats in the control group, supporting long-term efficacy [20]. There are a few potential explanations for this finding in our study. Uremic toxins display significant biological variability. Although every effort was made to obtain samples at a standard time of day to mitigate variability, the power may not have been adequate due to the limited number of cats available. Secondly, uremic toxins also accumulate in tissues, and there is a correlation between biofluid and tissue reduction in concentrations of IDS in response to the administration of AST-120 [24,25]. It could be theorized that uremic toxins may continue to leach out of tissues over time.

Our study aimed to assess the short-term effects of daily Renaltec administration in cats with IRIS Stages 2 and 3 eating the same diet and living in a similar housing situation, allowing a unique opportunity to mitigate variability. However, some of these aspects may limit the applicability of the study to the greater population of feline patients, particularly as it relates to diet. Many cats with CKD may rotate diets or eat small meals throughout the day. Interestingly, median serum concentrations of IDS (1922 ng/mL; range 1030–4876) were subjectively lower in the research cat population than those previously reported for client-owned cats with IRIS Stage 2 eating a variety of diets (3370 ng/mL; range 746–10,300) [2]. Both studies utilized the same analytical laboratory, and although not directly comparable for a number of reasons, these findings perhaps imply that good diet compliance may have contributed to relatively low baseline serum concentrations of IDS. This phenomenon was not observed with pCS.

Dose titration of oral adsorbents is recommended by some practitioners in human medicine and has been documented to be associated with a better outcome in human CKD patients [16]. Although there was a trend, our study did not find a significant difference in percent change in uremic toxins from baseline when SID and BID dosing were compared. However, only with BID dosing was the percent change in IDS and pCS serum concentrations significantly decreased in comparison to SOC. Although it is likely that this difference is attributable to a dose-related effect, this cannot be definitely concluded from this study design. Three changes were made during the BID Trial that could have affected these results: a change from once-daily to twice-daily feeding, a new diet formulation with added a prebiotic, and doubling the Renaltec dose. However, the data imply that administering Renaltec twice a day in cats that have twice-daily meals may maximize the benefit of therapy in some cats.

There was a statistically significant increase in serum creatinine and BUN concentrations over the 4-month course of the SOC and SID Trials, which is suggestive of progression of CKD. However, the degree of change (median BUN increased from 22 mg/dL to 24 mg/dL, and the median creatinine increased from 1.9 mg/dL to 2.1 mg/dL) could be considered clinically insignificant. A similar finding was not observed in the BID Trial, although this trial was of shorter duration, nor was a similar finding observed in the preliminary results of the 6-month trial of Renaltec in cats with Stages 2 and 3 of the disease [20]. Therefore, these results may be attributable to biologic or analytical variability. If progression did occur, the observation that IDS and pCS significantly decreased in the face of CKD progression potentially makes the change more meaningful and could be considered a positive outcome during the SID Trial.

The decrease in body weight found over the course of the SOC and SID Trials was unlikely to be clinically significant (median of 4.0 kg to 3.9 kg) as a whole, but significant weight loss occurred in individual cats, most commonly prior to the start of Renaltec administration. No significant change in body weight was found when just the period of Renaltec administration was considered. Body condition score and MCS did not significantly change over the course of the SOC and SID Trials. In the shorter time period of the BID Trial, weight, BCS, and MCS remained stable. All cats were reported to eat 100% of their food during all study phases. These results imply that a stabilization of weight loss occurred during the administration of Renaltec and may be important when taken in the broader context of the weight loss commonly associated with CKD [26]. A similar trend was observed in a study in client-owned cats with CKD where patients receiving placebo lost weight over the 6-month study period, whereas those who received Renaltec did not [20].

The potential improvement in serum bicarbonate concentrations and anion gap as a result of Renaltec administration was an unexpected finding. Metabolic acidosis is a common sequela of CKD and multifactorial in nature [27]. In broad terms, there is decreased acid excretion by the kidneys and increased endogenous acid production. Indoxyl sulfate has been described as a contributor to the development of metabolic acidosis [24]. Therefore, it is plausible that decreasing the systemic absorption of IDS and lessening the need for excretion would have a positive impact on acid–base status. Ultimately, this study was not designed to comprehensively evaluate why this occurred and these data are preliminary as power was not sufficient to draw definitive conclusions. The acid–base status of CKD patients is more accurately assessed by a urine ammonium–creatinine ratio as opposed to bicarbonate and anion gap concentrations [28]. The urine ammonium–creatinine ratio was not measured in this population of cats because an effect on acid–base status was not anticipated. Further studies are needed to evaluate if the improved acid–base status is repeatable and accurate, and further investigate the underlying mechanism of action.

Our study had several limitations. The sample size was limited to the size of the colony and this may have affected power. The studies were carried out in a sequential manner and the treatment groups were not randomized. The decision to perform the studies sequentially was made to decrease the possibility of error in medication administration and to also mitigate any possibility of a lag effect beyond the treatment period. As such, the study design did not allow for an assessment of whether effects on uremic toxins persisted beyond the study period, a phenomenon that was previously reported with AST-120 in cats [17]. An additional major limitation was that an inadvertent diet formulation change occurred at the start of the BID Trial. Therefore, while the study’s initial intent was to compare two dosing regimens, the diet formulation change may also have improved serum uremic toxin concentrations. Although similar in nutrient content, including protein and phosphorus (Table 1), the diet fed during the BID Trial contained betaine and a blend of prebiotic fibers designed to target uremic toxins [29]. Additionally, the protein source changed (chicken to oceanfish), which could have had an effect on uremic toxins. Lastly, the reference change values for the biological variability of uremic toxins that were utilized as a basis of comparison in this study were determined in normal cats eating a standardized diet [23], which may not be entirely translatable to a diseased population.

In conclusion, Renaltec decreased IDS and pCS serum concentrations in cats with experimentally induced CKD consuming a diet formulated for CKD. In addition, it may also improve acid–base status, but additional studies are needed to confirm and further evaluate these findings.

## 5. Conclusions

In conclusion, our data suggest that the daily administration of Renaltec can reduce serum concentrations of deleterious gut-derived uremic toxins in cats with CKD. No adverse effects of administration were appreciated in cats with IRIS Stage 2 and 3 experimentally induced CKD.

## Figures and Tables

**Table 1:** Formulation of kidney diets fed in Trials 1 and 2. Nutrients of concern are displayed as mg or g/100 kcal.

Diet	kcal/cup	Na (mg)	K (mg)	Protein (g)	Fat (g)	Phos (mg)	Ca (mg)	Crude Fiber (g)	TDF (g)	EPA + DHA (mg)
Hill’s Prescription Diet k/d w/Chicken (dry)	534	57	171	6.7	5.4	119	183	0.7	1.6	100
Hill’s k/d ACTIVBIOME+ Kidney Defense w/Oceanfish (dry)	498	59	172	6.8	5.2	118	183	0.7	1.5	135

TDF, total dietary fiber; EPA, eicosapentaenoic acid; DHA, docosahexaenoic acid.

**Table 2:** Descriptive statistics for selected parameters during the standard of care (SOC) and once-daily dosing (SID) Trials. Cats (n = 13) received SOC treatment for chronic kidney disease on SOC Days 0 through 56, and then SOC in addition to 500 mg of Renaltec orally SID on SID Days 0 through 56. Data are displayed as median (range).

	Laboratory Reference Range	SOC Day 0	SOC Day 28	SOC Day 56 (SID Day 0)	SID Day 28	SID Day 56
Weight ^a,b^ (kg)	-	4.0 (3.3–4.6)	3.9 (3–4.6)	3.9 (3.1–4.5)	3.9 (3–4.5)	3.8 (3.1–4.4)
BCS	-	6.0 (5–7)	5 (5–7)	5 (5–6)	5 (5–7)	5 (5–7)
MCS	-	1 (0–2)	1 (0–2)	1 (1–2)	1 (1–2)	1 (1–2)
HCT (%)	33.6–49	35.7 (28.2–43.6)	-	36.5 (27.2–40.1)	-	35.7 (27.4–39.5)
BUN ^b,d^ (mg/dL)	18–35	22 (18–35)	-	22 (17–37)	-	24 (20–44)
Creatinine ^b,d^ (mg/dL)	0.7–1.8	1.9 (1.5–4.0)	-	2.0 (1.6–4.0)	-	2.1 (1.6–4.7)
Phosphorous (mg/dL)	2.8–5.9	3.6 (2.8–4.4)	-	3.5 (2.4–4.5)	-	3.4 (2.4–4.6)
Bicarbonate (mmol/L)	15–23	16 (12–20)	-	16 (10–17)	-	17 (14–19)
Anion gap ^b,d^ (mmol/L)	19–29	22 (18–26)	-	23 (18–30)	-	20 (17–24)
Potassium (mmol/L)	3.6–5.3	4.4 (3.6–4.9)	-	4.6 (3.7–5.2)	-	4.6 (3.7–5.0)
UPC	<0.20	0.12 (0.02–0.20)	-	0.11 (0.05–0.30)	-	0.10 (0.02–0.50)
BP (mmHg)	100–160	156 (107–184)	-	142 (116–202)	-	137 (108–170)
IDS (ng/mL) ^c^	-	2012 (617–5827)	1780 (1015–4682)	1652 (1257–4119)	1417 (664–3856)	1513 (1157–5479)
pCS (ng/mL) ^c^	-	10,422 (5143–47,316)	8535 (308–37,815)	12,720 (268–34,609)	7604 (263–26,575)	8520 (290–34,114)
		**Average of SOC Days 0, 28, 56**			**Average of SID Days 28, 56**	
IDS (ng/mL)	-	1800 (1030–4876)			1592 (944–4669)	
pCS (ng/mL)	-	10,141 (1906–39,913)			6861 (277–30,345)	

Abbreviations: BCS, body condition score; MCS, muscle condition score; HCT, hematocrit; BUN, blood urea nitrogen; UPC, urine protein–creatinine ratio; BP, blood pressure; IDS, indoxyl sulfate; pCS, p-cresol sulfate. Superscript letters denote significant differences between time points (*p* < 0.05).^a^ SOC Day 0 to SOC Day 28. ^b^ SOC Day 0 vs. SOC Day 56. ^c^ SOC Day 56 vs. SID Day 28. ^d^ SOC Day 56 vs. SID Day 56.

**Table 3:** Descriptive statistics for selected measurements during the twice-daily (BID) Trial. Cats (n = 12) received 500 mg of Renaltec orally BID on BID Days 0 through 56. Data are displayed as median (range).

	Laboratory Reference Range	BID Day 0	BID Day 28	BID Day 56
Weight (kg)	-	3.8 (3.1–4.3)	3.9 (3.2–4.3)	3.9 (3.0–4.3)
BCS	-	5 (4–7)	5 (4–6)	5 (4–6)
MCS	-	2 (1–3)	2 (1–4)	2 (1–4)
HCT (%) ^b^	33.6–49.0	35.6 (28.2–41.1)	-	31.3 (25.5–37.4)
BUN (mg/dL) ^b^	18–35	24 (18–40)	-	26 (22–46)
Creatinine (mg/dL)	0.7–1.8	2.1 (1.5–4.4)	-	2.2 (1.5–4.2)
Phosphorous (mg/dL) ^b^	2.8–5.9	3.9 (2.4–4.8)	-	4.3 (3.6–6.0)
Bicarbonate (mmol/L) ^b^	15–23	15 (13–18)	-	16 (14–19)
Anion gap (mmol/L)	19–29	22 (18–29)	-	22 (18–25)
Potassium (mmol/L)	3.6–5.3	4.5 (3.7–5.0)	-	4.0 (3.7–5.2)
UPC	<0.20	0.10 (0–0.40)	-	0.10 (0.04–0.40)
BP (mmHg)	100–160	133 (108–157)	-	129 (104–173)
IDS (ng/mL) ^a,b^	-	1704 (1244–5666)	1369 (558–4465)	1426 (631–3874)
pCS (ng/mL) ^b^	-	11,839 (5250–45,684	5952 (2451–20,477)	7055 (2031–20,168)

Abbreviations: BCS, body condition score; MCS, muscle condition score; HCT, hematocrit; BUN, blood urea nitrogen; UPC, urine protein–creatinine ratio; BP, blood pressure; IDS, indoxyl sulfate; pCS, p-cresol sulfate. Superscript letters denote significant differences between time points (*p* < 0.05), ^a^ BID Day 0 vs. BID Day 28, ^b^ BID Day 0 vs. BID Day 56.

## Data Availability

The raw data supporting the conclusions of this article will be made available by the authors on request.
